# Circulation Patterns, Genetic Diversity, and Public Health Implications of Enterovirus D68, Europe, 2014–2024

**DOI:** 10.3201/eid3204.251022

**Published:** 2026-04

**Authors:** Cristina Andrés, Ignasi Prats-Méndez, Sofie Midgley, Natasa Berginc, Alejandra González-Sánchez, Caroline Klint Johannesen, Andrés Antón, Patricia Nadal-Barón, Thea K. Fischer, Heli Harvala, Kimberley S.M. Benschop

**Affiliations:** Vall d’Hebron Hospital Universitari, Vall d’Hebron Institut de Recerca (VHIR), Vall d’Hebron Barcelona Hospital Campus, Barcelona, Spain (C. Andrés, I. Prats-Méndez, A. González-Sánchez, A. Antón, P. Nadal-Barón); Statens Serum Institut, Copenhagen, Denmark (S. Midgley); National Laboratory of Health, Environment and Food, Laboratory for Polio, Ljubljana, Slovenia (N. Berginc); Nordsjællands Hospital, Hillerød, Denmark (C.K. Johannesen, T.K. Fischer); Centro de Investigación Biomédica en red de Enfermedades Infecciosas CIBERINFEC, Instituto Carlos III, Madrid, Spain (A. Antón); University of Copenhagen, Copenhagen (T.K. Fischer); National Health Service Blood and Transplant, London, UK (H. Harvala); University of Oxford, Oxford, UK (H. Harvala); Turku University Central Hospital, Turku, Finland (H. Harvala); University of Turku, Turku (H. Harvala); National Institute for Public Health and the Environment, Bilthoven, the Netherlands (K.S.M. Benschop)

**Keywords:** viruses, enterovirus, EV-D68, respiratory infections, genetic diversity, surveillance, Europe, genetic clades

## Abstract

Enterovirus D68 (EV-D68) represents a continuing public health concern, given its association with severe respiratory illness and neurologic complications. In this study, we analyzed EV-D68 circulation and genetic evolution during 2014–2024 using data from 18 countries in Europe. Of 61,297 enterovirus-positive specimens, molecular detection and viral protein 1 sequencing identified 3,541 (6%) EV-D68 cases. A biennial circulation pattern was observed; detection rates ranged from 9% in 2014 to 0.9% in 2019. The pattern was disrupted in 2020 because of measures implemented in response to the COVID-19 pandemic, but then notable increases occurred in 2021 (14%), 2022 (10.7%), and 2024 (20.6%). Subgenogroups B3 (59.8%) and A2/D (28.0%) were predominant; A2/D reemerged as dominant in 2024. Mutation analyses revealed changes in antigenic regions. Our findings underscore the persistent adaptation and resurgence of EV-D68 after COVID-19. Continued genomic surveillance is essential to monitor transmission patterns caused by antigenic changes.

Enterovirus D68 (EV-D68), a nonpolio enterovirus of the *Picornaviridae* family, has attracted attention because of its potential to cause severe respiratory illness and neurologic complications, particularly in children. First isolated in 1962, EV-D68 was sporadically detected until outbreaks in 2010 revealed its capacity for widespread transmission (*1*–*4*). Moreover, several clades (A–D) and subclades (A1, A2/D, B1, B2, B3, and C) have been described (*5*). Subsequent studies have also demonstrated that EV-D68 exhibits a cyclical pattern of circulation and a biennial recurrence (*6*,*7*), which continued until the COVID-19 pandemic, when the incidence of seasonal respiratory viruses was disrupted.

The ongoing circulation of EV-D68 poses a challenge for public health because outbreaks frequently coincide with increased rates of hospitalization for respiratory distress and, occasionally, cases of acute flaccid myelitis (AFM) (*8*). The neuropathogenic properties of the virus have been linked to specific amino acid residues. In particular, Leser et al. (*9*) recently identified 4 amino acid substitutions in the *VP1* gene (I553L, D554N, A650T, and K835E) that are associated with neurovirulence in mice. Several recent studies have shown that neutralizing antibody responses to EV-D68 increase with age and that infection with 1 clade can generate cross-reactive immunity against other clades, providing key insights into population immunity and transmission dynamics (*10*–*12*).

Despite improvements in surveillance efforts, comprehensive analysis of EV-D68 circulation patterns and the elucidation of viral features remains limited. This study provides a summary of trends in EV-D68 circulation in Europe during 2014–2024, particularly focusing on prevalence, genetic diversity, and potential public health implications.

## Methods

### Data Collection

We obtained epidemiologic and molecular data from the passive and hospital-based surveillance systems (enterovirus, influenza-like illness, or acute flaccid paralysis [AFP]) of various institutions affiliated with the European Non-Poliovirus Enterovirus Network through a data collection form (Appendix Table 1) and other studies (*1*,*3*,*13*–*16*). Specimens from patients with symptoms suggestive of an enterovirus-related acute respiratory infection or neurologic illness (meningitis, AFP, or myelitis) were tested for virological confirmation according to case definition (*17*). Information regarding the samples that were tested and subsequently identified as EV-D68 was gathered during October 2014–December 2024. We also extracted demographics and clinical data from previous published works (*1*,*3*,*13*–*16*). Institutional Review Board approval (PR(AG)419/2023) was obtained from the Hospital Universitari Vall d’Hebron Clinical Research Ethics Committee.

### Enterovirus Detection and Characterization

We conducted enterovirus detection using different real-time multiplex RT-PCRs (Table 1) (*34*). We subjected the partial viral protein (VP) 1 or the complete genome to sequencing for enterovirus typing (*35*) and genomic annotation of all enterovirus-positive specimens, as previously reported (*1*–*3*,*33*,*36*–*38*) (Table 1). We conducted further molecular characterization of EV-D68 by phylogenetic analyses using IQ-TREE multicore version 2.4.0 (Model Finder) (*39*) to define the genetic clades (Appendix Figure 1). References used for EV-D68 phylogeny at clade level are according to the following GenBank accession numbers: A1, KT959173; A2/D, F726085, KT959178, KU242683, and KY358058; B1, KP745751; B2, KP745768; B3, KT711083, KT803593, KU982558, and KY385886. In addition, we analyzed amino acid substitutions within antigenic epitopes of the VP1 protein, specifically the BC-loop (positions 642–655) and DE-loop (positions 692–698), and other areas recently linked to neuropathogenesis (*9*) using the Data Explorer module in MEGA6 (*40*). Numbering of amino acid positions in translated nucleotide sequences are relative to the Fermon strain (GenBank accession no. AY426531) and clinical isolate U.S./IL/14-18952 (GenBank accession no. KM851230) for the sequence annotation of immunogenicity and neuropathogenic sites. We determined the frequency of amino acid residues per antigenic site using EV-D68 A2/D and B3 clades, compared between prepandemic (2014–2019), pandemic (2020–2022), and postpandemic (2023–2024) periods, and plotted with Weblogo 3 (*41*). We compiled GenBank accession numbers of sequences used from the previously published studies and newly generated sequences (Appendix Table 2).

**Table 1:** Details of participant countries in study of circulation patterns, genetic diversity, and public health implications of EV-D68 circulation, Europe, 2014–2024

Country	No. institutions	Screening methods	Sequencing method references	No. EV-D68 positive/no. enterovirus positive (%)†
Austria	1	NA	(*18*)	7/625 (1)
Belgium	2	Feces: enterovirus PCR on GI-TAC assay; respiratory samples: enterovirus PCR and EV-D68 PCR on respiratory TAC assay; others: in-house enterovirus PCR; CSF: FilmArray panel (BioFired Diagnostics https://www.biofiredx.com)	NA	158/3,281 (5)
Bulgaria	1	Cell culture and inhouse enterovirus PCR	NA	0/61 (0)
Croatia	1	NA	NA	9/1,066 (1)
Czech Republic	1	Cell culture and in-house enterovirus PCR	NA	0/310 (0)
Denmark	1	In-house enterovirus and RV PCR (*19*)	(*18*)	293/6,204 (5)
Estonia	1	NA	NA	0/27 (0)
Finland	3	In-house enterovirus and RV PCR	(*18*)	100/1,177 (8)
France	1	In-house enterovirus, rhinovirus, and EV-D68 PCR (*20*)	(*18*)	549/10,111 (5)
Germany	4	In-house enterovirus and RV PCR	(*21*)	46/3,237 (1)
Hungary	1	In-house enterovirus PCR (*22*)	(*18*)	0/112 (0)
Iceland	1	In-house enterovirus and EV-D68 PCR (*23*,*24*)	(*25*,*26*)	13/219 (6)
Ireland	1	Respiratory samples: Luminex NxTAG Respiratory Panel (EV/RV) (Diasorin, https://www.diasorin.com); enterovirus (*18*) and in-house EV-D68 PCR	(*18*)	6/552 (1)
Italy	2	Allplex Respiratory Panel (Seegene, https://www.seegene.com) and in-house enterovirus and EV-D68 PCR (*27*,*28*)	(*18*)	129/1,696 (8)
Latvia	1	NA	NA	0/104 (0)
Lithuania	1	NA	NA	0/22 (0)
Luxembourg	1	NA	NA	1/346 (0)
Netherlands	8	In-house enterovirus and EV-D68 PCR (*13*, *29*, *30*)	(*18*)	236/5,747 (4)
Norway	2	In-house enterovirus and EV-D68 PCR (*31*)	(*18*)	89/1,048 (8)
Poland	1	NA	NA	0/159 (0)
Portugal	1	Allplex Respiratory Panel (Seegene)	NA	23/95 (24)
Romania	1	NA	NA	9/9 (100)
Slovakia	1	NA	NA	0/130 (0)
Slovenia	3	In-house enterovirus and EV-D68 PCR (*23*)	(*18*)	119/1,502 (8)
Spain	4	Respiratory: Allplex Respiratory Panel (Seegene); feces and CSF: RealCycle EV/hPeV detection (Progenie) and Allplex Meningitis V2 Panel (Seegene)	(*18*, *32*, *33*)	1,354/9,721 (14)
Sweden	1	Allplex Respiratory Panel (Seegene) and in-house enterovirus and EV-D68 PCR (*27*)	(*18*)	234/4,188 (6)
United Kingdom	4	In-house enterovirus and EV-D68 PCR	(*18*); Colindale reference laboratory sequencing	166/9,548 (2)
Total	50			3,541/61,297 (6)

*CSF, cerebrospinal fluid; EV-D68, enterovirus D68; GI-TAC, Gastrointestinal TaqMan Array Card; hPeV, human parechovirus; NA, not applicable. †Studies used in number of enterovirus-positive specimens: (*1*,*3*,*13*–*16*).

## Results

### EV-D68 Circulation through Europe

**Figure 1:**
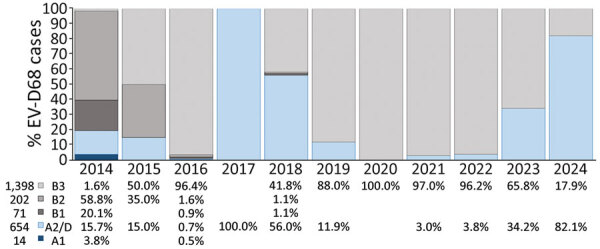
Yearly percentage and global distribution of EV-D68 cases, by clade, in study of circulation patterns, genetic diversity, and public health implications of EV-D68 circulation, Europe, 2014–2024. Numbers at bottom left indicate total numbers of cases by clade during the study period. EV-D68, enterovirus D68.

During October 2014–December 2024, a total of 61,297 specimens from 50 institutions across 18 countries in Europe (Table 1) were laboratory-confirmed as enterovirus. A total of 3,541 (6%) of 61,297 were identified as EV-D68; of those, 2,339 (66%) were further genetically characterized based on the VP1 sequence (n = 2,057) or complete genome (n = 282). Subgenogroups B3 (1,398 [59.8%]) and A2/D (654 [28.0%]) were overall the most prevalent, followed by B2 (202 [8.6%]), B1 (71 [3.0%]), and A1 (14 [0.6%]) (Figure 1; Appendix Figure 1). EV-D68 was predominantly collected from persons with acute respiratory infection symptoms, primarily children; incidence among adolescents and adults (>15 years of age) increased during 2024 (Tables 2, 3).

**Table 2:** Age groups of case-patients available from study of circulation patterns, genetic diversity, and public health implications of enterovirus D68 circulation, Europe, 2014–2024*

Age group, y	No. (%)			
	2014	2015–2017	2018–2023	2024
<1	120 (**30.85**)	103 (**32.29**)	259 (21.48)	22 (4.37)
1–5	135 (**34.70**)	143 (**44.83**)	569 (**47.18**)	86 (17.10)
6–15	56 (14.40)	35 (10.97)	154 (12.77)	54 (10.74)
>15	78 (20.05)	38 (11.91)	224 (18.57)	341 (**67.79**)

*Bold indicates most affected populations.

**Table 3:** Clinical data available from cases in study of circulation patterns, genetic diversity, and public health implications of enterovirus D68 circulation, Europe, 2014–2024*

Symptom	Global no. (%)
Respiratory	657 (91.50)
Gastrointestinal	–
Neurologic	38 (5.29)
Hand, foot, and mouth disease/skin	–
Fever	14 (1.95)
Other	9 (1.25)

*Dash indicates there were no cases in which those symptoms were present.

**Figure 2:**
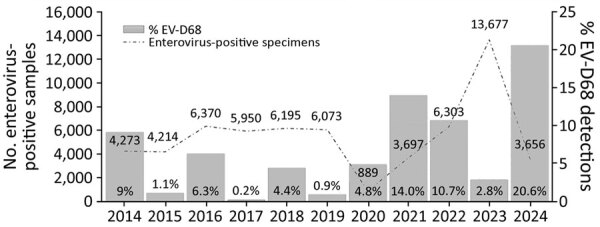
Yearly distribution of enterovirus-positive specimens and percentage of EV-D68–related infections in study of circulation patterns, genetic diversity, and public health implications of EV-D68 circulation, Europe, 2014–2024. EV-D68, enterovirus D68.

In Europe, EV-D68 circulation was predominantly observed during even years (9% in 2014, 6.3% in 2016, 4.4% in 2018), although circulation was disrupted in 2020 because of the implementation of preventive measures for SARS-CoV-2 (Figure 2). Nevertheless, we observed a notable upsurge in EV-D68 cases in 2021 (14.0%) across multiple countries (Figure 2). We also observed subsequent circulation in 2022 (10.7%) and during the 2024 season (20.6%). Although fewer cases were detected in 2023 (2.8%), the numbers were still substantially higher than in the low-circulation years before the pandemic (1.1% in 2015, 0.2% in 2017, and 0.9% in 2019) (Figure 2). Regardless of the changing epidemiology of EV-D68 over the years, we observed further levels of predominance of the EV-D68 clades over the study period (Figure 1). Although B3 prevailed in 2015 and 2016, A2/D dominated in 2017; both subgenogroups cocirculated in 2018. B3 was dominant again in the following years but decreased in 2024, when A2/D was the dominant subgenogroup (82.1%) (Figure 1).

### Mutation Analyses of EV-D68 Strains

#### Immunogenic Epitopes

**Figure 3:**
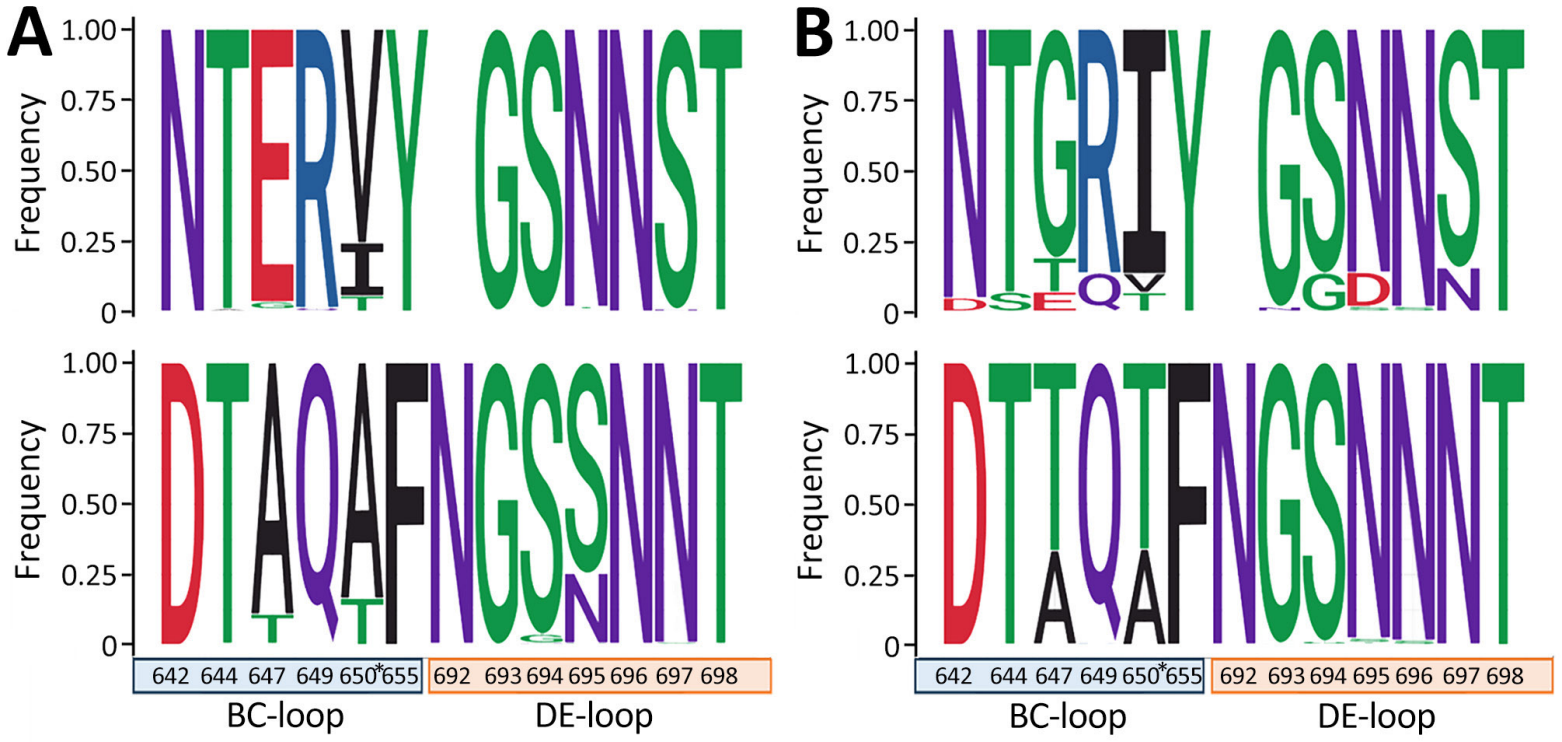
Frequency of amino acid changes within the antigenic epitopes of the viral protein 1 (BC- and DE-loops) in clades A2/D and B3 in study of circulation patterns, genetic diversity, and public health implications of enterovirus D68 circulation, Europe, 2014–2024. A) Amino acid changes in the prepandemic period (2014–2019) in clade A2/D (top) and clade B3 (bottom); B) changes during pandemic and postpandemic periods (2020–2024) in clade A2/D (top) and clade B3 (bottom). Amino acids are colored according to their chemistry. Numbering is related to complete enterovirus D68 genome. Asterisks indicate position 650, also related to neurovirulence in mice. Image was created with WebLogo 3 (*41*).

We have provided the frequency of amino acid changes detected at antigenic sites (BC- and DE-loops) of EV-D68 strains in prepandemic, pandemic, and postpandemic periods (Figure 3), as well as the complete mutation analysis of *VP1* gene per year and per clade (Appendix Figures 2, 3). We observed a clear change in amino acid diversity in both A2/D (Appendix Figure 2) and B3 (Appendix Figure 3) clades during the 2 periods. Positions 647 and 650 in the BC-loop exhibited the greatest degree of variation compared with other sites in both clades (Figure 3). Within the A2/D clade, prepandemic sequences were predominantly characterized by glutamic acid at position 647 (E647) and valine at position 650 (V650). Although the isoleucine at position 650 (I650) dominated until 2016, a transition to valine (V650) was observed in 2017 and 2018; the detection rate during 2019 was 50% (Appendix Figure 2). Conversely, during the pandemic and postpandemic era (2020–2024), we observed a marked shift in residue predominance. Glycine at position 647 (G647) and isoleucine at position 650 (I650) became the dominant residues, largely replacing the prepandemic amino acids and indicating a substantial change in the antigenic landscape of the BC-loop (Figure 3; Appendix Figures 2, 4). Regarding the B3 clade, prepandemic sequences exhibited a high predominance of alanine at both position 647 (A647) and 650 (A650). In the aftermath of 2020, those residues underwent a substantial replacement by threonine at both positions (T647 and T650), which subsequently became predominant in postpandemic B3 sequences. In addition, within the DE-loop, position 695 exchange of amino acids before and after the pandemic (from S695 to N695) also indicates antigenic drift in this loop (Figure 3; Appendix Figures 3, 4).

#### Neurovirulent Epitopes

We further investigated the genetic diversity of the recently identified neurovirulence-associated markers within the *VP1* gene (I553L, D554N, A650T, and K835E) (Table 4; Appendix Figure 4) (*9*). In the context of the A2/D clade, we observed no substantial changes among prepandemic, pandemic, and postpandemic periods; we observed neurovirulent amino acid changes at L553 and E835 and a glutamic acid at position 554 predominantly identified, in contrast to position 650, which was described previously as changing from a valine to an isoleucine (Table 4). With regard to the B3 clade, L553 was only identified in 2014, albeit in 1 sequence, whereas E554 was only identified in 2023–2024 in a shift from D to E. As described previously, we observed an apparent change to the neurovirulent threonine at position 650 (T650). In addition to A2/D, the neurovirulent glutamic acid at position 835 (E835) was detected throughout the decade (Table 4).

**Table 4:** Amino acid changes within mice-neurovirulent-related sites (553, 554, 650, and 835) in clades A2/D and B3 in study of circulation patterns, genetic diversity, and public health implications of enterovirus D68 circulation, Europe, 2014–2024*

Year	No. sequences (WG)		Clade		EV-D68 complete genome numeration											
					553	554		650		835	No. sequences (WG)	Clade	553	554	650	835
					VP1 region								VP1 region			
					1	2		BC† 98		283			1	2	BC† 98	283
					I	D		A		K			I	D	A	K
2014	49		A2/D		L	E		I		E	5	B3	L/V	•	•	E
2015	3		A2/D		–	–		I		–	10 (2)	B3	•	•	•	E
2016	3 (1)		A2/D		L	E		I		E	424 (10)	B3	•	•	•	E
2017	5		A2/D		–	–		V		E	–	B3	–	–	–	–
2018	209 (32)		A2/D		L	E		V		E	156 (43)	B3	•	•	•	E
2019	8 (4)		A2/D		L	E		V		E	59 (13)	B3	•	•	T	E
2020	–		A2/D		–	–		–		–	13 (8)	B3	•	•	T	E
2021	15 (3)		A2/D		L	E		I		E	481 (44)	B3	•	•	T	E
2022	6 (3)		A2/D		L	E		I/V		E	150 (65)	B3	•	•	T	E
2023	13 (5)		A2/D		L	E		I		E	25 (12)	B3	•	E	T	E
2024	343 (28)		A2/D		L	E		I		E	75 (9)	B3	•	E	T	E
0%–25%		26%–49%		50%–74%			75%–100%									

*Percentages are based on partial (650) or whole (553, 554, and 835) genome sequences. Numbering is related to complete EV-D68 genomes. Dots indicate amino acid is the same as in reference sequence. Dashed line notes start of COVID-19 pandemic period. A, alanine; E, glutamic acid; EV-D68, enterovirus D68; I, isoleucine; I/V, isoleucine/valine; L, leucine; L/V, leucine/valine; T, threonine; WG, whole genome; –, no information on amino acid position. †Refers to antigenic epitope BC-loop.

## Discussion

This study offers a comprehensive overview of the circulation of EV-D68 across Europe over a decade, emphasizing its prevalence, seasonality, and evolution. Our findings underscore the persistent adaptation and resurgence of EV-D68 in the postpandemic era.

Our analysis revealed a biennial seasonal pattern of EV-D68 activity and a pronounced prevalence during even years. That pattern is consistent with the findings of previous studies that have documented similar biennial trends in North America (*42*) and Europe (*3*,*33*,*36*,*38*). The interruption of the pattern in 2020 corroborates findings from other investigations that reported a reduced circulation of respiratory pathogens during the COVID-19 pandemic because of the implementation of preventive measures against SARS-CoV-2 (*43*). The resurgence of EV-D68 in 2021 and the subsequent peaks in 2022 and 2024 lend further support to the hypothesis that relaxing public health restrictions led to the reestablishment of EV-D68 transmission chains. Whether transmission reverts to a biennial cycle and changes in genetic diversity remain will require continued surveillance. Moreover, the predominance of EV-D68–related respiratory infections, especially in children, is cause for concern, particularly in the context of AFM. Although initial epidemiologic studies indicated a temporal association between EV-D68 outbreaks and AFM cases, subsequent neuropathological evidence demonstrating EV-D68 within anterior horn motor neurons has directly supported a causal relationship (*2*,*44*,*45*).

The high prevalence of EV-D68 in the 2024 season in some countries in Europe (20.6%) contrasts with earlier reports from the 2014 outbreak in Europe and United States, in which prevalence reached ≈9%–12% (*19*,*37*). That observation underscores the potential for variations in outbreak magnitude, which might be influenced by factors such as population immunity, viral evolution, public health interventions, and enhanced surveillance systems, particularly after the COVID-19 pandemic, which could have improved the detection of several respiratory viruses, such as EV-D68.

Genetic characterization of EV-D68 specimens revealed some replacements and dynamic genetic drifts in the predominance of subgenogroups B3 and A2/D over time, as has been reported in other regions and with similar yearly trends to this study (*36*,*46*,*47*). The exchange of clades might be associated with antigenic drift and immune selection pressures, which could have also driven the reemergence of A2/D as the dominant clade (82.1%) during 2024 with additional substitutions within the antigenic epitopes, as we observed. Moreover, that exchange could be related to differences in age distribution among B3- and A2/D-related cases, as previously described (*36*). During 2024, according to the available data, the ratio A2/D:B3 exhibited a higher prevalence among adults than in children (Table 2). That phenomenon could be attributed to immune evasion in adults, which is likely caused by changes in antigenic properties caused by mutations in antigenic epitopes, resulting in reduced neutralization by underlying neutralizing antibodies (*36*). Regarding that phenomenon, we observed amino acid changes within the *VP1* gene, particularly in the BC-loop and DE-loop in positions 647, 650 (A2/D and B3), and 695 (B3), which mirror mutations reported during earlier outbreaks (*36*,*47*). Those changes could be associated with potential changes in viral fitness, neuropathogenesis, and immune evasion, as previously reported (*9*,*48*), or could represent an adaptive shift influencing receptor-binding properties and host-immune interactions (*49*). The significance of the identified changes, along with the changes seen over time in the BC-loop and DE-loop, require further investigations.

In addition to mutations in the VP1 region, changes outside the antigenic sites, such as the mice-neurovirulent E835 mutation, also deserve attention. E835 has been identified as a key factor in increasing neurotropism in mice, potentially promoting viral replication in motor neurons and enhancing retrograde axonal transport (*9*). In our series, E835 was mostly observed (instead of K835) in A2/D and B3. Also, the L553 mutation has been frequently reported in A2/D-related cases during the study period, which is also suspected to be a neurovirulence-related change in mice. Conversely, the B3 clade appears to have retained the isoleucine variant at this position, which is characterized as nonvirulent (*15*), yet we observed a shift to the neurovirulent threonine at position 650 after 2020. We observed a similar pattern at site 554, where there is a tendency to shift toward glutamic acid (E), another supposedly nonvirulent substitution seen in both the A2/D and B3 clades, although that residue position might also play a role in immune escape (*15*). The presumed neurovirulent genetic markers were identified in some A2/D- and B3-related cases in this study, but no increase in AFM-related cases was detected (*8*). Further research is required to ascertain whether those mutations or combinations of them are associated with severe clinical outcomes.

Of note, the emergence of SARS-CoV-2 coincided with increased mutational events on EV-D68, suggesting that competition with other respiratory pathogens during the pandemic might have driven such gradual changes (*50*). The circulation of new lineages that might have acquired an evolutionary advantage, possibly through immune evasion mechanisms (changes within antigenic epitopes), could then explain the predominance of the A2/D subgenogroup in 2024. Given the biennial epidemic cycles of EV-D68 and its apparent resurgence after the COVID-19 pandemic, global genomic monitoring is increasingly needed to identify emerging variants that could result in higher incidence and increased risk for neurologic complications. Resurgence shows an opportunity for targeted public health interventions, such as enhancing diagnostic capacity during anticipated peaks and promoting preventive measures.

Although this study provides valuable insights and is supported by both existing literature and recent findings, its first limitation is its reliance on laboratory-confirmed cases, which might potentially underestimate the true burden of EV-D68, because milder cases frequently go undiagnosed. In addition, although surveillance data might not be uniformly collected across Europe (and are largely unavailable at that level), the relevance of this study lies in the participation of 50 institutions from 18 countries in Europe. In addition, the limited number of fully genetically characterized cases might fail to capture the full diversity of circulating strains, at least during the 2024 season. Future studies should prioritize expanding genomic surveillance and integrating serologic data to better understand the effects of EV-D68 in both community and hospital populations, in relation to population-level immunity against this virus.

In conclusion, the reemergence of EV-D68 after the COVID-19 pandemic and its genetic evolution highlight current limitations that might pose a substantial public health challenge. An enhanced surveillance system based on real-time genomic monitoring of circulating viruses, coupled with a deeper understanding of the factors driving the evolution of this reemerging pathogen, is crucial to mitigating its impact.

Appendix Additional information about circulation patterns, genetic diversity, and public health implications of enterovirus D68 circulation, Europe, 2014–2024.
