# Supplementary material for: Circulation Patterns, Genetic Diversity, and Public Health Implications of Enterovirus D68, Europe, 2014–2024
Source: Emerg Infect Dis. 2026 Apr;32(4):491–9. doi: 10.3201/eid3204.251022 (PMC13094849; doi:10.3201/eid3204.251022)
Supplement: Appendix — Additional information about circulation patterns, genetic diversity, and public health implications of enterovirus D68 circulation, Europe, 2014–2024. [file 25-1022-Techapp-s1.pdf]

Article DOI: https://doi.org/10.3201/eid3204.251022

EID cannot ensure accessibility for supplementary materials supplied by authors. Readers who have difficulty accessing supplementary content should contact the authors for assistance.

Circulation Patterns, Genetic Diversity, and Public Health Implications of Enterovirus D68, Europe, 2014–2024

Appendix

Appendix Table 1. Data collection form ENPEN during 2024

COUNTRY	MONTH / YEAR	EV +	EV-D68	CLADE A2	CLADE B3
Netherlands	jan-24	33	0	0	0
Netherlands	feb-24	43	0	0	0
Netherlands	march-24	55	0	0	0
Netherlands	apr-24	65	0	0	0
Netherlands	may-24	99	0	0	0
Netherlands	june-24	102	0	0	0
Netherlands	july-24	185	1	N/A	N/A
Netherlands	aug-24	133	3	2	1
Netherlands	sep-24	116	11	9	2
Netherlands	oct-24	110	13	7	6
Netherlands	nov-24	65	5	3	2
Netherlands	dic-24	22	0	0	0
Slovenia	jan-24	13	0	N/A	N/A
Slovenia	feb-24	6	0	N/A	N/A
Slovenia	march-24	1	0	N/A	N/A
Slovenia	apr-24	3	0	N/A	N/A
Slovenia	may-24	5	0	N/A	N/A
Slovenia	june-24	8	0	N/A	N/A
Slovenia	july-24	3	0	N/A	N/A
Slovenia	aug-24	2	0	N/A	N/A
Slovenia	sep-24	11	10	N/A	N/A
Slovenia	oct-24	25	17	N/A	N/A
Slovenia	nov-24	9	1	N/A	N/A
Slovenia	dic-24	3	0	N/A	N/A
Denmark	jan-24	54	0	0	0
Denmark	feb-24	31	0	0	0
Denmark	march-24	84	0	0	0
Denmark	apr-24	90	0	0	0
Denmark	may-24	70	0	0	0
Denmark	june-24	105	0	0	0
Denmark	july-24	145	0	0	0
Denmark	aug-24	143	8	1	2
Denmark	sep-24	277	85	20	36
Denmark	oct-24	213	64	5	22
Denmark	nov-24	194	25	5	8
Denmark	dic-24	96	6	4	2
Spain	jan-24	17	0	0	0
Spain	feb-24	38	1	1	0
Spain	march-24	31	0	0	0
Spain	apr-24	25	0	0	0
Spain	may-24	42	0	0	0
Spain	june-24	51	0	0	0
Spain	july-24	37	2	0	2
Spain	aug-24	73	28	13	15
Spain	sep-24	168	141	17	124
Spain	oct-24	241	179	35	144
Spain	nov-24	175	88	5	83
Spain	dic-24	70	50	6	44

*N/A, not available.

Appendix Table 2. GenBank Ids used in this work and new sequences generated during the study period

GenBank_Ids											Newly generated sequences					
KM887894.1	KP830130.1	KY272923.1	MH307388.1	MN403127.1	MN403273.1	MT795862.1	OM831168.1	OQ139602.1	OQ933694.1	OR365021.1	NL24-1001-01	101668028	102418359	161516035	198310418	560047070
KM887895.1	KP830131.1	KY272924.1	MH307389.1	MN403128.1	MN403274.1	MT795863.1	OM831169.1	OQ139603.1	OQ933695.1	OR365022.1	NL24-1002-01	101668515	102419120	161518859	198310419	560047076
KM887896.1	KP830132.1	KY272925.1	MH307390.1	MN403129.1	MN403275.1	MT795864.1	OM831170.1	OQ139604.1	OQ933696.1	OR365023.1	NL24-1014-01	101671510	102419490	161519194	198310420	560047119
KM887897.1	KP830133.1	KY272926.1	MH307391.1	MN403130.1	MN403276.1	MT795865.1	OM831171.1	OQ139605.1	OQ933697.1	OR365024.1	NL24-1045-01	101672397	102419823	161519234	198310426	560047273
KM887898.1	KP830134.1	KY272927.1	MH307392.1	MN403131.1	MN403277.1	MT795866.1	OM831172.1	OQ139606.1	OQ933698.1	OR365025.1	NL24-1060-01	101672416	102420039	161519266	198310428	560047277
KM887899.1	KP830135.1	KY272928.1	MH307393.1	MN403132.1	MN403278.1	MT795867.1	OM831173.1	OQ139607.1	OQ933699.1	OR365026.1	NL24-1064-01	101672441	102420135	161519274	198310624	560047472
KM887900.1	KP830136.1	KY272929.1	MH307394.1	MN403133.1	MN403279.1	MT795868.1	OM831174.1	OQ139608.1	OQ933700.1	OR365027.1	NL24-1088-01	101672504	102421196	161519279	198310627	560047521
KM887901.1	KT220441.1	KY272930.1	MH307395.1	MN403134.1	MN403280.1	MW731816.1	OM831175.1	OQ139609.1	OQ933701.1	OR365028.1	NL24-789-01	101673138	102421620	161519280	198310629	560047527
KM887902.1	KT220442.1	KY272931.1	MH307396.1	MN403135.1	MN403281.1	MW731817.1	OM831176.1	OQ139610.1	OQ933702.1	OR365029.1	NL24-976-01	101673158	102423069	161519372	198310630	560048050
KM887903.1	KT220443.1	KY272932.1	MH307397.1	MN403136.1	MN403282.1	MW731818.1	OM831177.1	OQ139611.1	OQ933703.1	OR365030.1	NL24-990-01	101673460	102423612	161519373	198310828	560048506
KM887904.1	KT220444.1	KY272933.1	MH307398.1	MN403137.1	MN403283.1	MW731819.1	OM831178.1	OQ139612.1	OQ933704.1	OR365031.1	100096404	101673696	102423874	170114950	198311044	560049157
KM887905.1	KT220445.1	KY272934.1	MH307399.1	MN403138.1	MN403284.1	MW731820.1	OM831179.1	OQ139613.1	OQ933705.1	OR365032.1	100322871	101673842	102424131	170128873	198311059	560049416
KM887906.1	KT220446.1	KY272935.1	MH307400.1	MN403139.1	MN403285.1	MW731821.1	OM831180.1	OQ139614.1	OQ933706.1	OR365033.1	100330479	101673973	102424193	170130380	198311065	560049419
KM924544.1	KT220447.1	KY272936.1	MH307401.1	MN403140.1	MN403286.1	MW731822.1	OM831181.1	OQ139615.1	OQ933707.1	OR365034.1	100331543	101674160	102424584	170140878	198311068	589912034
KM924545.1	KT220448.1	KY272937.1	MH307402.1	MN403141.1	MN403287.1	MW731823.1	OM831182.1	OQ139616.1	OQ933708.1	PP548243.1	100332347	101675168	102426500	170141090	198311336	741065295
KM924546.1	KT220449.1	KY272938.1	MH307403.1	MN403142.1	MN403288.1	MW731824.1	OM831183.1	OQ139617.1	OQ933709.1	PP548244.1	100336145	101675849	102426740	170141332	198311380	741078178
KM924547.1	KT220450.1	KY272939.1	MH307404.1	MN403143.1	MN403289.1	MW731825.1	OM831184.1	OQ139618.1	OQ933710.1	PP548245.1	100345758	101675910	102427057	170141336	198311381	741078461
KP153538.1	KT220451.1	KY272940.1	MH307405.1	MN403144.1	MN403290.1	MW731826.1	OM831185.1	OQ139619.1	OQ933711.1	PP548246.1	100347262	101677651	102427203	170141340	198311382	741079070
KP153539.1	KT220452.1	KY272941.1	MH307406.1	MN403145.1	MN403291.1	MW731827.1	OM831186.1	OQ139620.1	OQ933712.1	PP548247.1	100349487	101677658	102427363	170141950	198311383	741079152
KP153540.1	KT220453.1	KY272942.1	MH307407.1	MN403146.1	MN403292.1	MW731828.1	OM831187.1	OQ139621.1	OQ933713.1	PP548248.1	100372634	101677706	102428584	170143046	198311384	741079701
KP153541.1	KT220454.1	KY272943.1	MH885651.1	MN403147.1	MN403293.1	MW731829.1	OM831188.1	OQ139622.1	OQ933714.1	PP947790.1	100384985	101679012	102428792	170143330	198311388	741080363
KP153542.1	KT220455.1	KY272944.1	MK105983.1	MN403148.1	MN403294.1	MW731830.1	OM831189.1	OQ139623.1	OQ933715.1	PP947791.1	100394749	101679080	102429489	170145160	198311537	741080956
KP153543.1	KT220456.1	KY272945.1	MK105984.1	MN403149.1	MN403295.1	MW731831.1	OM831190.1	OQ139624.1	OQ933716.1	PP947792.1	100409872	101679339	102430072	171618815	198311538	741081029
KP153544.1	KT220457.1	KY272946.1	MK105985.1	MN403150.1	MN403296.1	MW731832.1	OM831191.1	OQ139625.1	OQ933717.1	PP947793.1	100516315	101679534	102430510	171619445	198311541	770307829
KP153545.1	KT220458.1	KY272947.1	MK105986.1	MN403151.1	MN403297.1	MW731833.1	OM831192.1	OQ139626.1	OQ933718.1	PP947794.1	100527806	101679650	102430904	171619590	198311542	770312429
KP153546.1	KT220459.1	KY272948.1	MK105987.1	MN403152.1	MN403298.1	MW731834.1	OM831193.1	OQ139627.1	OQ933719.1	PP947795.1	100529549	101680587	102430944	171620096	198311545	
KP189392.1	KT220460.1	KY272949.1	MK105988.1	MN403153.1	MN403299.1	MW731835.1	OM831194.1	OQ139628.1	OR179039.1	PP947796.1	100701425	101680865	102431059	171621545	198311546	
KP189393.1	KT220461.1	KY272950.1	MK105989.1	MN403154.1	MN726756.1	MW731836.1	OM831195.1	OQ139629.1	OR179040.1	PP947797.1	100884735	101681289	102431375	171622432	198311547	
KP189394.1	KT220462.1	KY272951.1	MK121710.1	MN403155.1	MN726757.1	MW731837.1	OM831196.1	OQ139630.1	OR179041.1	PP947798.1	100890111	101682613	102431494	171763459	198311548	
KP189395.1	KT220463.1	KY272952.1	MK121711.1	MN403156.1	MN726758.1	MW731838.1	OM831197.1	OQ148174.1	OR179042.1	PP947799.1	100894812	101682615	102431713	171765494	198311549	
KP189396.1	KT220464.1	KY272953.1	MK121712.1	MN403157.1	MN726759.1	MW731839.1	OM831198.1	OQ148347.1	OR179043.1	PP947800.1	100903144	101682819	102432018	172305042	198311550	
KP189397.1	KT220465.1	KY272954.1	MK121713.1	MN403158.1	MN726760.1	MW731840.1	OM831199.1	OQ148348.1	OR179044.1	PQ414985.1	100904638	101683245	102432423	172305044	198311551	
KP189398.1	KT220466.1	KY272955.1	MK121714.1	MN403159.1	MN726761.1	MW731841.1	OM831200.1	OQ148349.1	OR179045.1	PQ414986.1	100915119	101683328	102432939	172305057	198311554	
KP189399.1	KT220467.1	KY272956.1	MK121715.1	MN403160.1	MN726762.1	MW731842.1	OM831201.1	OQ148350.1	OR179046.1	PQ414987.1	100922910	101683811	102433311	172305104	198311559	
KP189400.1	KT220468.1	KY272957.1	MK121716.1	MN403161.1	MN726763.1	MW731843.1	OM831202.1	OQ148351.1	OR179047.1	PQ414988.1	100924948	101684746	102433694	172305336	198311671	
KP189401.1	KT220469.1	KY272958.1	MK121717.1	MN403162.1	MN726764.1	MW731844.1	OM831203.1	OQ148352.1	OR179048.1	PQ414989.1	100927737	101685121	102435103	172305337	198311766	
KP189402.1	KT220470.1	KY272959.1	MK121718.1	MN403163.1	MN726765.1	MW731845.1	OM831204.1	OQ148353.1	OR179049.1	PQ414990.1	100936895	101685665	102435581	172305440	198311767	

GenBank_Ids											Newly generated sequences				
KP189403.1	KT220471.1	KY272960.1	MK121719.1	MN403164.1	MN726766.1	MW731846.1	OM831205.1	OQ148354.1	OR179050.1	PQ414991.1	101622192	101685902	102435659	172309343	198311768
KP196362.2	KT220472.1	LN681316.2	MK121720.1	MN403165.1	MN726767.1	MW731847.1	OM831206.1	OQ148355.1	OR179051.1	PQ414992.1	101622195	101686086	102435773	172309417	198311977
KP196363.2	KT220473.1	LN681317.2	MK121721.1	MN403166.1	MN726768.1	MW731848.1	OM831207.1	OQ148356.1	OR179052.1	PQ414993.1	101622275	101686180	102435813	172313620	198311978
KP196364.2	KT220474.1	LN681318.2	MK121722.1	MN403167.1	MN726769.1	MW731849.1	OP267493.1	OQ148357.1	OR179053.1	PQ414994.1	101634809	101687184	102436099	172313656	198311983
KP196365.2	KT220475.1	LN681319.2	MK121723.1	MN403168.1	MN726770.1	MW731850.1	OP267494.1	OQ148358.1	OR179054.1	PQ414995.1	101635153	101687589	102436227	172313729	198311984
KP196366.2	KT220476.1	LN681320.2	MK121724.1	MN403169.1	MN726771.1	MW731851.1	OP267495.1	OQ148359.1	OR179055.1	PQ414996.1	101635221	101688212	102436360	172313803	198311989
KP196367.2	KT220477.1	LN681321.2	MK121725.1	MN403170.1	MN726772.1	MW731852.1	OP267496.1	OQ148360.1	OR179056.1	PQ414997.1	101637449	101689059	102436569	172313816	198311990
KP196368.2	KT220478.1	LN681322.2	MK121726.1	MN403171.1	MN726773.1	MW731853.1	OP267497.1	OQ148361.1	OR179057.1	PQ414998.1	101638617	101689374	102436999	172314114	198312017
KP196369.2	KT220479.1	LN681323.2	MK121727.1	MN403172.1	MN726774.1	MW731854.1	OP267498.1	OQ160429.1	OR179058.1	PQ414999.1	101638649	101689479	102437032	198046412	198312869
KP196370.1	KT220480.1	LN681324.2	MK121728.1	MN403173.1	MN726775.1	MW731855.1	OP267499.1	OQ160430.1	OR179059.1	PQ415000.1	101638775	101690189	102437864	198105756	198313099
KP196371.2	KT220481.1	LN681325.2	MK121729.1	MN403174.1	MN726776.1	MW731856.1	OP267500.1	OQ160431.1	OR179060.1	PQ415001.1	101640193	101690259	102439430	198257316	198313100
KP196372.2	KT220482.1	LN681326.2	MK121730.1	MN403175.1	MN726777.1	MW731857.1	OP267501.1	OQ160432.1	OR179061.1	PQ415002.1	101640361	101691140	102440397	198281210	198313159
KP196373.2	KT220483.1	LN681327.2	MK301336.1	MN403176.1	MN726778.1	MW731858.1	OP267502.1	OQ160433.1	OR179062.1	PQ415003.1	101640363	101691165	102441817	198281817	198313164
KP196374.2	KT220484.1	LN681328.2	MK301337.1	MN403177.1	MN726779.1	MW731859.1	OP267503.1	OQ259308.1	OR179063.1	PQ415004.1	101640414	101692566	102443293	198292370	198313165
KP196375.1	KT220485.1	LN681329.2	MK301338.1	MN403178.1	MN726780.1	MW731860.1	OP267504.1	OQ259309.1	OR179064.1	PQ415005.1	101640472	101692713	102443322	198303091	198313170
KP196376.2	KT220486.1	LN681330.2	MK301339.1	MN403179.1	MN726781.1	MW731861.1	OP267505.1	OQ259310.1	OR179065.1	PQ415006.1	101640971	101695074	102444999	198303092	198313171
KP196377.1	KT220487.1	LN681331.2	MK301340.1	MN403180.1	MN726782.1	MW731862.1	OP267506.1	OQ259311.1	OR179066.1	PQ415007.1	101641130	101695144	102446755	198306190	198313172
KP196378.1	KT220488.1	LN681332.2	MK301341.1	MN403181.1	MN726783.1	MZ576283.1	OP267507.1	OQ259312.1	OR179067.1	PQ415008.1	101641490	101695663	102449522	198306191	198313182
KP307989.1	KT220489.1	LN681333.2	MK301342.1	MN403182.1	MN726784.1	MZ576284.1	OP267508.1	OQ259313.1	OR179068.1	PQ415009.1	101641548	101696690	102455692	198306425	198313186
KP307990.1	KT220490.1	LN681334.2	MK301343.1	MN403183.1	MN726785.1	MZ576285.1	OP267509.1	OQ259314.1	OR179069.1	PQ415010.1	101642056	101698335	102459761	198306625	198313190
KP307991.1	KT220491.1	LN681335.2	MK301344.1	MN403184.1	MN726786.1	MZ576286.1	OP267510.1	OQ259315.1	OR179070.1	PQ415011.1	101642112	101700962	102460030	198306858	198313341
KP307992.1	KT220492.1	LN681336.2	MK301345.1	MN403185.1	MN726787.1	MZ576287.1	OP267511.1	OQ259316.1	OR179071.1	PQ415012.1	101642221	101702209	102462055	198307036	198313344
KP406467.1	KT220493.1	LN681337.2	MK301346.1	MN403186.1	MN726788.1	MZ576288.1	OP267512.1	OQ259317.1	OR179072.1	PQ415013.1	101642222	101703002	102471449	198307232	198313378
KP406468.2	KT220494.1	LN681338.2	MK301347.1	MN403187.1	MN726789.1	MZ576289.1	OP267513.1	OQ259318.1	OR179073.1	PQ415014.1	101643020	101703617	102479206	198307234	198313387
KP406469.2	KT220495.1	LN681339.2	MK301348.1	MN403188.1	MN726790.1	MZ576290.1	OP267514.1	OQ259319.1	OR179074.1	PQ415015.1	101644761	101704123	102484459	198307236	198313389
KP406470.2	KT220496.1	LN681340.2	MK301349.1	MN403189.1	MN726791.1	MZ576291.1	OP267515.1	OQ259320.1	OR179075.1	PQ415016.1	101644867	101705899	110070195	198307619	198313390
KP406471.1	KT220497.1	LN874222.1	MK301350.1	MN403190.1	MN726792.1	MZ576292.1	OP267516.1	OQ285848.1	OR179076.1	PQ415017.1	101644919	101709278	110073590	198307623	198313393
KP406472.2	KT220498.1	LN874223.1	MK301351.1	MN403191.1	MN726793.1	MZ576293.1	OP267517.1	OQ285849.1	OR179077.1	PQ415018.1	101645019	101716301	110075023	198307719	198313396
KP406473.2	KT220499.1	LN874224.1	MK301352.1	MN403192.1	MN726794.1	MZ576294.1	OP267518.1	OQ285850.1	OR179078.1	PQ415019.1	101645141	101717705	110240902	198307720	198313636
KP406474.1	KT220500.1	LN874225.1	MK301353.1	MN403193.1	MN726795.1	MZ576295.1	OP267519.1	OQ285851.1	OR179079.1	PQ415020.1	101645458	101718107	110277653	198307721	198313638
KP406475.1	KT220501.1	LN874226.1	MK301354.1	MN403194.1	MN726796.1	MZ576296.1	OP267520.1	OQ285852.1	OR179080.1	PQ415021.1	101645675	101720181	110333205	198307722	198313707
KP406476.1	KT220502.1	LN874227.1	MK301355.1	MN403195.1	MN726797.1	MZ576297.1	OP267521.1	OQ285853.1	OR179081.1	PQ415022.1	101645791	101731892	110334552	198307724	198313708
KP406477.1	KT220503.1	LN874228.1	MN245396.1	MN403196.1	MN726798.1	MZ576298.1	OP267522.1	OQ285854.1	OR179082.1	PQ415023.1	101646627	101775547	110338371	198307725	198313712
KP406478.1	KT220504.1	LN874229.1	MN245397.1	MN403197.1	MN726799.1	MZ576299.1	OP267523.1	OQ586762.1	OR179083.1	PQ415024.1	101646993	101815886	110349332	198307764	198313715
KP406479.2	KT220505.1	LN874230.1	MN245398.1	MN403198.1	MN726800.1	MZ576300.1	OP267524.1	OQ586763.1	OR179084.1	PQ415025.1	101647229	101818056	110354551	198307978	198313717
KP406480.1	KU886245.1	LN874231.1	MN245399.1	MN403199.1	MN726801.1	MZ576301.1	OP267525.1	OQ586764.1	OR179085.1	PQ415026.1	101647282	101874839	110369082	198308064	198313718
KP406481.1	KU886246.1	LN874232.1	MN245401.1	MN403200.1	MN764886.1	MZ576302.1	OP267526.1	OQ586765.1	OR179086.1	PQ415027.1	101647350	101893885	110369818	198308065	198313731
KP406482.1	KU886247.1	LN874233.1	MN245402.1	MN403201.1	MN764887.1	MZ576303.1	OP267527.1	OQ586766.1	OR179087.1	PQ415028.1	101647657	101902345	110528088	198308083	198313746
KP406483.1	KU886248.1	LN874234.1	MN245403.1	MN403202.1	MN764888.1	MZ576304.1	OP267528.1	OQ586767.1	OR179088.1	PQ415029.1	101647889	101903563	110554340	198308085	198313747
KP406484.1	KU886249.1	LN874235.1	MN245404.1	MN403203.1	MN764889.1	MZ576305.1	OP267529.1	OQ586768.1	OR179089.1	PQ415030.1	101648152	101906774	110676702	198308086	198313749
KP406485.1	KX685066.1	LN874236.1	MN245405.1	MN403204.1	MN809623.1	MZ576306.1	OP267530.1	OQ586769.1	OR179090.1	PQ415031.1	101648646	101907651	110686709	198308087	198313750
KP406486.2	KX685067.1	LN874237.1	MN245406.1	MN403205.1	MN809624.1	MZ576307.1	OP267531.1	OQ586770.1	OR179091.1	PQ415032.1	101649973	101909739	110691119	198308088	198313751
KP406487.2	KX685068.1	LN874238.1	MN245407.1	MN403206.1	MN809625.1	MZ576308.1	OP267532.1	OQ586771.1	OR179092.1	PQ415033.1	101650244	101911444	110692594	198308262	198313753
KP406488.2	KX685069.1	LN874239.1	MN245408.1	MN403207.1	MN809626.1	MZ576309.1	OP267533.1	OQ586772.1	OR179093.1	PQ415034.1	101650772	101912271	110693059	198308341	198313755
KP406489.1	KX685070.1	LN874240.1	MN245409.1	MN403208.1	MN814240.1	MZ576310.1	OP267534.1	OQ586773.1	OR179094.1	PQ415035.1	101650789	101914256	110693239	198308342	198314157
KP406490.1	KX685071.1	LN874241.1	MN245410.1	MN403209.1	MN814241.1	MZ576311.1	OP267535.1	OQ586774.1	OR179095.1	PQ415036.1	101651394	101916637	110693436	198308343	198314165
KP406491.1	KX685072.1	LN874242.1	MN245411.1	MN403210.1	MN814242.1	MZ576312.1	OP389245.1	OQ586775.1	OR179096.1	PQ415037.1	101651482	101919777	110693577	198308345	198314167
KP406492.1	KX685073.1	LN874243.1	MN245412.1	MN403211.1	MN814243.1	MZ576313.1	OP389246.1	OQ586776.1	OR179097.1	PQ415038.1	101651920	101920637	110693995	198308346	198314609
KP406493.1	KX685074.1	LN874244.1	MN245413.1	MN403212.1	MN814244.1	MZ576314.1	OQ120627.1	OQ586777.1	OR179098.1	PQ415039.1	101651923	101921049	110694442	198308347	198314723

**GenBank_Ids**											**Newly generated sequences**				
KP406494.1	KX685075.1	LN874245.1	MN245414.1	MN403213.1	MN814245.1	MZ576315.1	OQ120628.1	OQ586778.1	OR179099.1	PQ415040.1	101651948	101921862	110694684	198308403	198314729
KP406495.2	KX685076.1	LN874246.1	MN245415.1	MN403214.1	MN814246.1	MZ576316.1	OQ120629.1	OQ586779.1	OR179100.1	PQ415041.1	101651988	101923805	110694764	198308405	199820228
KP406496.1	KX685077.1	LN874247.1	MN245416.1	MN403215.1	MN814247.1	MZ576317.1	OQ120630.1	OQ586780.1	OR179101.1	PQ415042.1	101652001	101924690	110695100	198308407	199820567
KP657737.1	KX685078.1	LN874248.1	MN245417.1	MN403216.1	MN814248.1	MZ576318.1	OQ120631.1	OQ586781.1	OR179102.1	PQ415043.1	101652016	101932954	110695109	198308408	199823301
KP657738.1	KX685079.1	LN874249.1	MN245418.1	MN403217.1	MN814249.1	MZ576319.1	OQ139546.1	OQ586782.1	OR179103.1	PQ415044.1	101652092	101933733	110695256	198308512	199823354
KP657739.1	KX685080.1	LN874250.1	MN245419.1	MN403218.1	MN814250.1	MZ576320.1	OQ139547.1	OQ586783.1	OR179104.1	PQ415045.1	101652260	101935750	110695637	198308514	199823749
KP657740.1	KX685081.1	LN874251.1	MN245420.1	MN403219.1	MN814251.1	MZ576321.1	OQ139548.1	OQ586784.1	OR179105.1	PQ415046.1	101652932	101937040	110695774	198308517	199823780
KP657741.1	KX685082.1	LN874252.1	MN245421.1	MN403220.1	MN832496.1	MZ576322.1	OQ139549.1	OQ586785.1	OR179106.1	PQ415047.1	101653183	101938061	110696215	198308921	199823781
KP657742.1	KX685083.1	LN874253.1	MN245422.1	MN403221.1	MN832497.1	MZ576323.1	OQ139550.1	OQ586786.1	OR179107.1	PQ415048.1	101653473	101938455	110696784	198309129	222310450
KP657743.1	KX685084.1	LT745914.1	MN245423.1	MN403222.1	MN832498.1	MZ576324.1	OQ139551.1	OQ586787.1	OR179108.1	PQ426627.1	101654458	101938480	110697604	198309130	242211902
KP657744.1	KX710328.1	LT745915.1	MN245424.1	MN403223.1	MN832499.1	MZ576325.1	OQ139552.1	OQ586788.1	OR179109.1	PQ426628.1	101654721	101938581	110699953	198309131	250833268
KP657745.1	KX830887.1	MF073335.1	MN245425.1	MN403224.1	MN832500.1	MZ576326.1	OQ139553.1	OQ586789.1	OR179110.1	PQ426629.1	101654772	101940972	110702163	198309132	251095871
KP657746.1	KX830888.1	MF073336.1	MN245426.1	MN403225.1	MN896973.1	MZ576327.1	OQ139554.1	OQ586790.1	OR179111.1	PQ426630.1	101655105	101941649	110702558	198309326	251096211
KP657747.1	KX830889.1	MF073337.1	MN245427.1	MN403226.1	MN896974.1	MZ576328.1	OQ139555.1	OQ586791.1	OR179112.1	PQ426631.1	101655136	101941655	110702628	198309328	251096220
KP725220.1	KX830890.1	MF073338.1	MN245428.1	MN403227.1	MN896975.1	MZ576329.1	OQ139556.1	OQ586792.1	OR179113.1	PQ426632.1	101655268	101942516	110704346	198309329	252730001
KP725221.1	KX830891.1	MF073339.1	MN245429.1	MN403228.1	MN896976.1	MZ576330.1	OQ139557.1	OQ586793.1	OR179114.1	PQ426633.1	101655561	101942642	110704394	198309330	252730009
KP725222.1	KX830892.1	MF073340.1	MN245430.1	MN403229.1	MN896977.1	MZ576331.1	OQ139558.1	OQ586794.1	OR179115.1	PQ426634.1	101655726	101943189	110705608	198309331	252731561
KP728259.1	KX830893.1	MF073341.1	MN245431.1	MN403230.1	MN896980.1	MZ576332.1	OQ139559.1	OQ586795.1	OR179116.1	PQ426635.1	101655859	101944632	110706897	198309332	252798273
KP744824.1	KX830894.1	MF073342.1	MN245432.1	MN403231.1	MN896981.1	MZ576333.1	OQ139560.1	OQ586796.1	OR179117.1	PQ426636.1	101655874	101946042	110707500	198309333	252798929
KP744825.1	KX830895.1	MF073343.1	MN245433.1	MN403232.1	MN896982.1	MZ576334.1	OQ139561.1	OQ586797.1	OR179118.1	PQ426637.1	101655918	101947138	110752021	198309334	252798940
KP744826.1	KX830896.1	MF073344.1	MN245434.1	MN403233.1	MN896983.1	MZ576335.1	OQ139562.1	OQ586798.1	OR179119.1	PQ426638.1	101655970	101947205	111225609	198309335	252798941
KP744827.1	KX830897.1	MF073345.1	MN245435.1	MN403234.1	MN896984.1	MZ576336.1	OQ139563.1	OQ586799.1	OR179120.1	PQ426639.1	101656063	101947348	111234685	198309336	252799249
KP744828.1	KX830898.1	MF073346.1	MN245436.1	MN403235.1	MN896985.1	MZ576337.1	OQ139564.1	OQ586800.1	OR179121.1	PQ426640.1	101656539	101948400	111258979	198309337	252799592
KP744829.1	KX830899.1	MF073347.1	MN245437.1	MN403236.1	MN896986.1	MZ576338.1	OQ139565.1	OQ586801.1	OR179122.1	PQ426641.1	101657035	101948730	111421410	198309340	510711315
KP744830.1	KX830900.1	MF073348.1	MN245438.1	MN403237.1	MN935869.1	MZ576339.1	OQ139566.1	OQ586802.1	OR179123.1	PQ426642.1	101658111	101949354	111425031	198309342	510802928
KP744831.1	KX830901.1	MF073349.1	MN245439.1	MN403238.1	MN935870.1	MZ576340.1	OQ139567.1	OQ586803.1	OR179124.1	PQ426643.1	101658635	101949370	111428504	198309407	510815193
KP744832.1	KX830902.1	MF073350.1	MN245440.1	MN403239.1	MT789734.1	MZ576341.1	OQ139568.1	OQ586804.1	OR179125.1	PQ426644.1	101658947	101949703	111808751	198309527	510827977
KP744833.1	KX830903.1	MF073351.1	MN245441.1	MN403240.1	MT789735.1	MZ576342.1	OQ139569.1	OQ933661.1	OR179126.1	PQ426645.1	101659567	101949964	111811292	198309528	510828304
KP744834.1	KX830904.1	MF073352.1	MN245442.1	MN403241.1	MT789736.1	MZ576343.1	OQ139570.1	OQ933662.1	OR179127.1	PQ426646.1	101661078	101951705	111817170	198309531	531011611
KP744835.1	KX830905.1	MF073353.1	MN245443.1	MN403242.1	MT789737.1	MZ576344.1	OQ139571.1	OQ933663.1	OR179128.1	PQ426647.1	101661316	101952818	111818854	198309533	531024424
KP744836.1	KX830906.1	MF073354.1	MN245444.1	MN403243.1	MT789738.1	MZ576345.1	OQ139572.1	OQ933664.1	OR179129.1	PQ426648.1	101661567	101953643	111823154	198309534	531024655
KP744837.1	KX830907.1	MH084317.1	MN245445.1	MN403244.1	MT789739.1	MZ576346.1	OQ139573.1	OQ933665.1	OR179130.1	PQ426649.1	101661624	101954485	111823848	198309535	531024734
KP744838.1	KX830908.1	MH084320.1	MN245446.1	MN403245.1	MT789740.1	MZ576347.1	OQ139574.1	OQ933666.1	OR179131.1	PQ426650.1	101661754	101959076	111828308	198309634	531025016
KP744839.1	KX830909.1	MH084323.1	MN245447.1	MN403246.1	MT789741.1	MZ576348.1	OQ139575.1	OQ933667.1	OR179132.1	PQ426651.1	101662008	101961229	111853165	198309635	531025318
KP745729.1	KX830910.1	MH084328.1	MN245448.1	MN403247.1	MT789742.1	MZ576349.1	OQ139576.1	OQ933668.1	OR179133.1	PQ426652.1	101662261	101963882	131092492	198309636	531025325
KP745730.2	KX830911.1	MH118296.1	MN253123.1	MN403248.1	MT789743.1	MZ576350.1	OQ139577.1	OQ933669.1	OR179134.1	PQ426653.1	101662433	101963941	131094705	198309637	531026380
KP745731.2	KX830912.1	MH138302.1	MN403103.1	MN403249.1	MT789744.1	MZ576351.1	OQ139578.1	OQ933670.1	OR179135.1	PQ426654.1	101662566	102158038	131105489	198309845	531026381
KP745732.2	KX830913.1	MH307365.1	MN403104.1	MN403250.1	MT789745.1	MZ576352.1	OQ139579.1	OQ933671.1	OR179136.1		101662681	102165339	131112004	198309847	531026384
KP745733.2	KX830914.1	MH307366.1	MN403105.1	MN403251.1	MT789746.1	OL829841.1	OQ139580.1	OQ933672.1	OR179137.1		101663275	102178517	131380705	198309848	531027356
KP745734.2	KX830915.1	MH307367.1	MN403106.1	MN403252.1	MT789747.1	OL829842.1	OQ139581.1	OQ933673.1	OR179138.1		101663375	102179359	131975314	198309903	534158547
KP745735.2	KX830916.1	MH307368.1	MN403107.1	MN403253.1	MT789748.1	OL829843.1	OQ139582.1	OQ933674.1	OR179139.1		101663388	102181721	132341093	198309904	534158562
KP745736.2	KX830917.1	MH307369.1	MN403108.1	MN403254.1	MT789749.1	OL829844.1	OQ139583.1	OQ933675.1	OR365002.1		101663621	102188280	132341155	198309905	534159204
KP745737.1	KX830918.1	MH307370.1	MN403109.1	MN403255.1	MT789750.1	OL829845.1	OQ139584.1	OQ933676.1	OR365003.1		101664380	102190853	132950329	198309906	534160531
KP745738.2	KX830919.1	MH307371.1	MN403110.1	MN403256.1	MT789751.1	OL829846.1	OQ139585.1	OQ933677.1	OR365004.1		101664395	102197729	150195966	198309908	534160771
KP745739.1	KX830920.1	MH307372.1	MN403111.1	MN403257.1	MT789752.1	OL829847.1	OQ139586.1	OQ933678.1	OR365005.1		101664707	102202886	150195968	198309909	534161168
KP745740.2	KX830921.1	MH307373.1	MN403112.1	MN403258.1	MT789753.1	OM811651.1	OQ139587.1	OQ933679.1	OR365006.1		101664965	102210558	150195970	198309910	554902362
KP745741.2	KX830922.1	MH307374.1	MN403113.1	MN403259.1	MT789754.1	OM811652.1	OQ139588.1	OQ933680.1	OR365007.1		101665932	102379314	150196670	198309911	554902373
KP745742.2	KX830923.1	MH307375.1	MN403114.1	MN403260.1	MT789755.1	OM831155.1	OQ139589.1	OQ933681.1	OR365008.1		101665965	102398767	150197341	198309912	554902376
KP745743.2	KX830924.1	MH307376.1	MN403115.1	MN403261.1	MT791927.1	OM831156.1	OQ139590.1	OQ933682.1	OR365009.1		101666091	102403906	150198342	198309913	554902378

GenBank_Ids										Newly generated sequences				
KP830119.1	KX830925.1	MH307377.1	MN403116.1	MN403262.1	MT791928.1	OM831157.1	OQ139591.1	OQ933683.1	OR365010.1	101666638	102405090	151056351	198309914	554902380
KP830120.1	KX830926.1	MH307378.1	MN403117.1	MN403263.1	MT791929.1	OM831158.1	OQ139592.1	OQ933684.1	OR365011.1	101666716	102408151	151106532	198309915	554902382
KP830121.1	KX830927.1	MH307379.1	MN403118.1	MN403264.1	MT791930.1	OM831159.1	OQ139593.1	OQ933685.1	OR365012.1	101666909	102409491	151194358	198309923	554908022
KP830122.1	KX830928.1	MH307380.1	MN403119.1	MN403265.1	MT791931.1	OM831160.1	OQ139594.1	OQ933686.1	OR365013.1	101666998	102410171	151195226	198310084	554908025
KP830123.1	KX830929.1	MH307381.1	MN403120.1	MN403266.1	MT791932.1	OM831161.1	OQ139595.1	OQ933687.1	OR365014.1	101667010	102414307	151195659	198310150	554908026
KP830124.1	KY272917.1	MH307382.1	MN403121.1	MN403267.1	MT791933.1	OM831162.1	OQ139596.1	OQ933688.1	OR365015.1	101667332	102414347	160136551	198310362	554908032
KP830125.1	KY272918.1	MH307383.1	MN403122.1	MN403268.1	MT791934.1	OM831163.1	OQ139597.1	OQ933689.1	OR365016.1	101667342	102414598	160273861	198310363	560046137
KP830126.1	KY272919.1	MH307384.1	MN403123.1	MN403269.1	MT795858.1	OM831164.1	OQ139598.1	OQ933690.1	OR365017.1	101667645	102414900	160859100	198310412	560046899
KP830127.1	KY272920.1	MH307385.1	MN403124.1	MN403270.1	MT795859.1	OM831165.1	OQ139599.1	OQ933691.1	OR365018.1	101667775	102415430	161512148	198310413	560046939
KP830128.1	KY272921.1	MH307386.1	MN403125.1	MN403271.1	MT795860.1	OM831166.1	OQ139600.1	OQ933692.1	OR365019.1	101667802	102415780	161512164	198310414	560046945
KP830129.1	KY272922.1	MH307387.1	MN403126.1	MN403272.1	MT795861.1	OM831167.1	OQ139601.1	OQ933693.1	OR365020.1	101667898	102417977	161512426	198310415	560047068

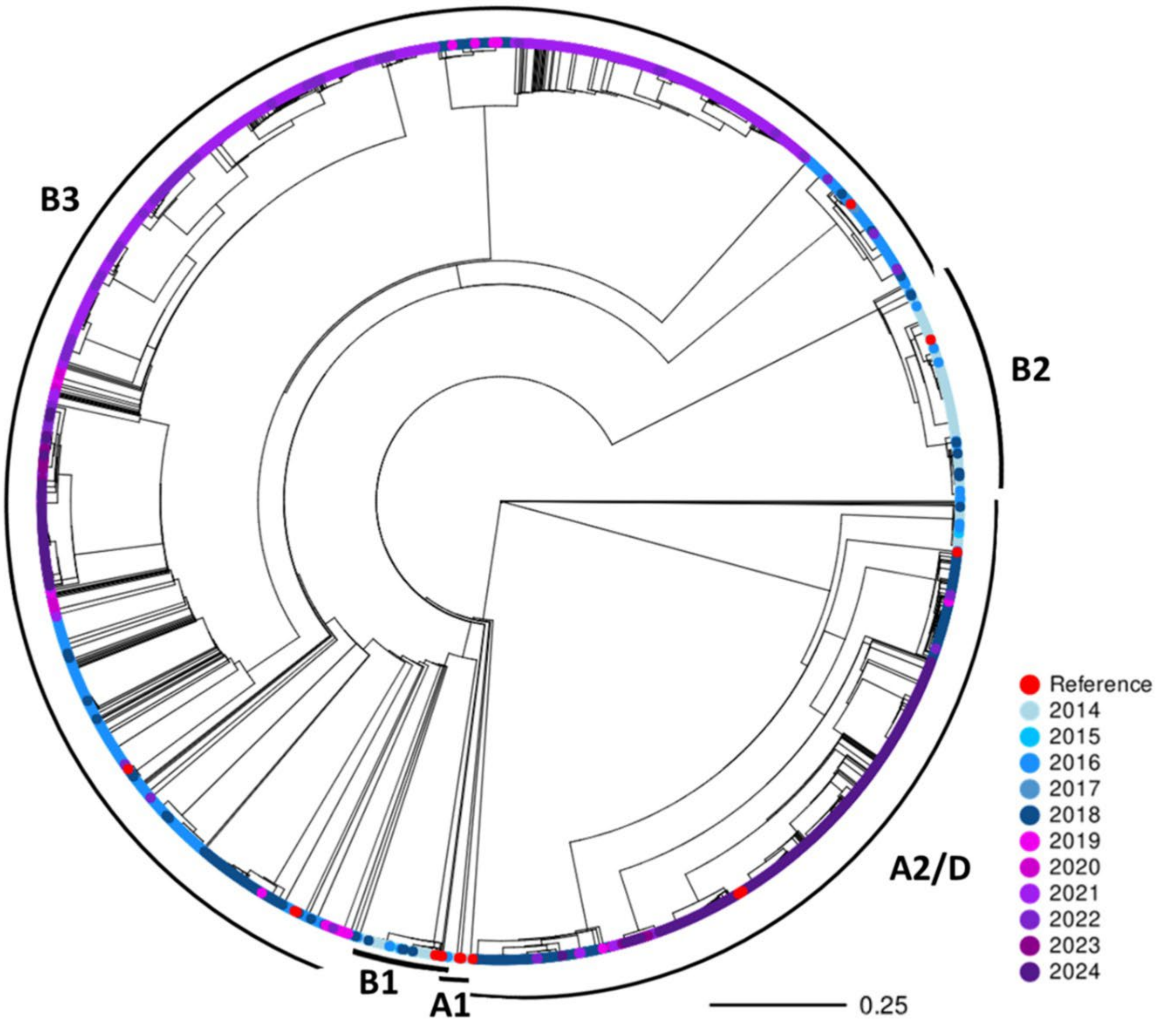

Appendix Figure 1. Evolutionary analysis of EV-D68 VP1 sequences from cases studied between 2014 and 2024. Phylogeny constructed by Maximum Likelihood method with a bootstrap value of 1000. Labels correspond to reference sequences in red and the year of detected cases.

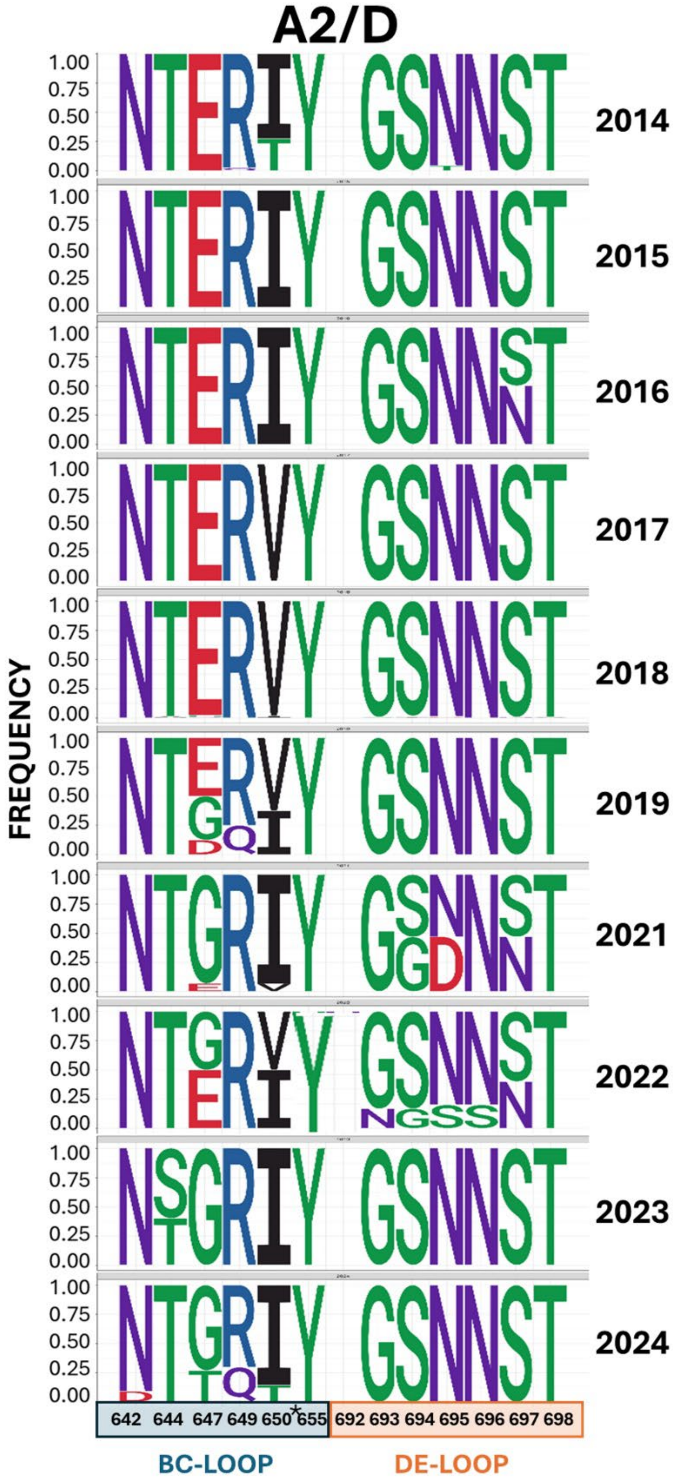

Appendix Figure 2. Frequency of amino acid changes within the antigenic epitopes of the VP1 (BC- and DE-loops) in clade A2/D during the study period created with Weblogo 3 (*1*). Amino acids are colored according to their chemistry. Numbering is related to complete EV-D68 genome. Position 650, also related to mice-neurovirulence, is labeled with an asterisk.

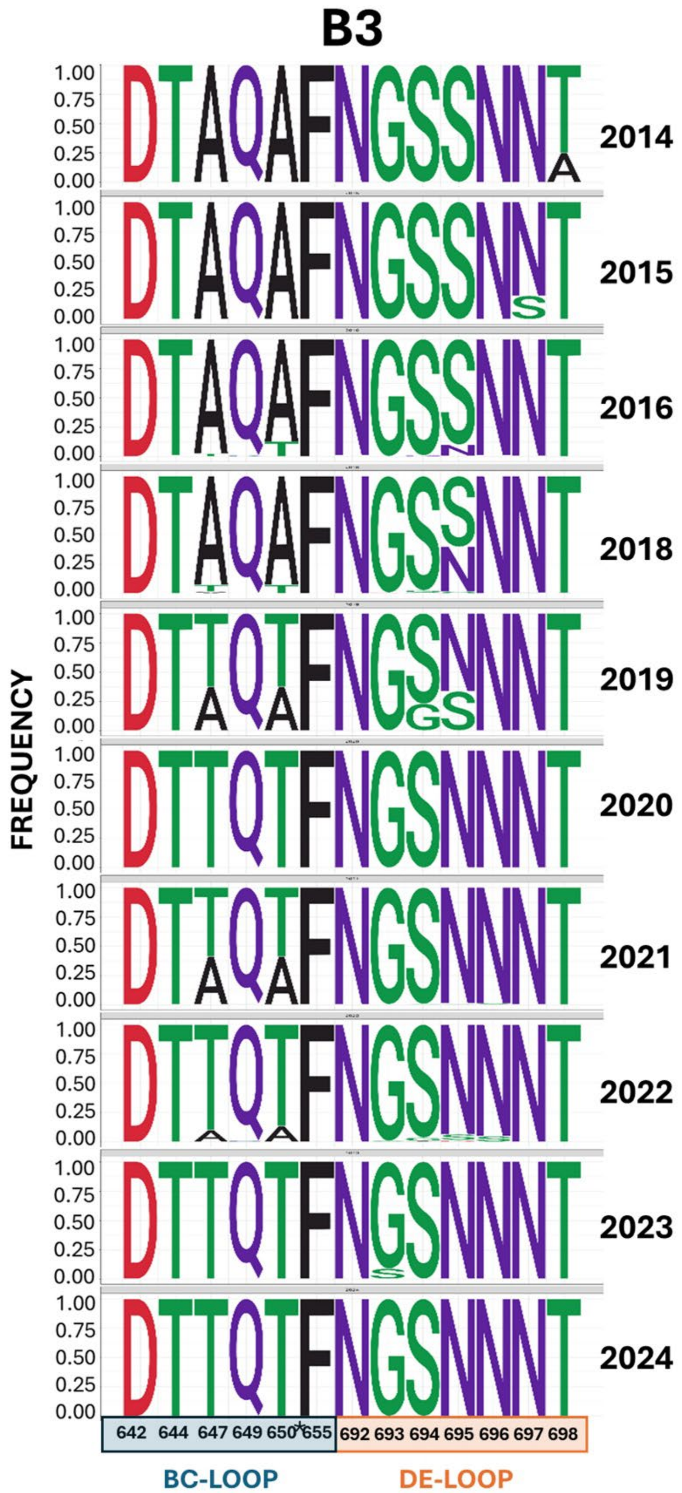

Appendix Figure 3. Frequency of amino acid changes within the antigenic epitopes of the VP1 (BC- and DE-loops) in clade B3 during the study period created with Weblogo 3 (*1*). Amino acids are colored according to their chemistry. Numbering is related to complete EV-D68 genome. Position 650, also related to mice-neurovirulence, is labeled with an asterisk.

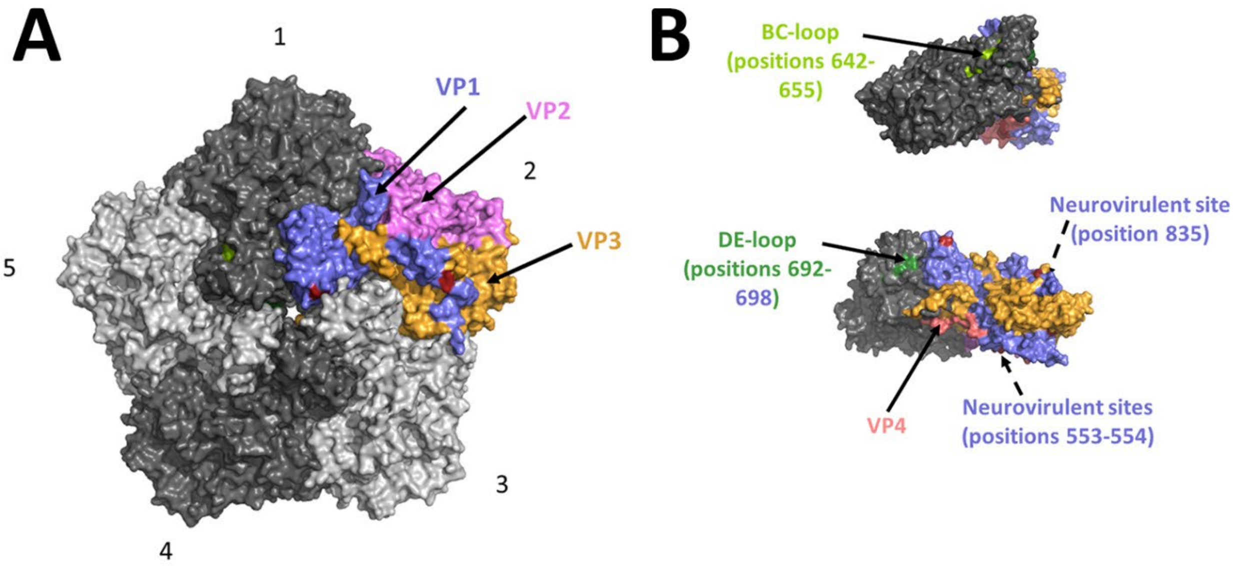

Appendix Figure 4. (A) Representation of the 5-fold symmetric arrangement (pentamer) of the crystal structure of the capsid protomer, which consists of VP1, VP2, VP3 and VP4. The five subunits are labeled by numbers 1–5. Subunit 2 shows the exposed proteins in purple (VP1), pink (VP2), bright orange (VP3) and red those amino acid changes observed. **(B)** Cross-section of the pentamer to distinguish better the antigenic epitopes (BC- and DE-loops), neurovirulent sites (internal positions – dotted arrow) and VP4 in light pink. Elaborated with Pymol following the structure 4WM8 from Liu et al. Science 2015 (*2*).

References

1. Crooks GE, Hon G, Chandonia JM, Brenner SE. WebLogo: a sequence logo generator. Genome Res. 2004;14:1188–90. PubMed https://doi.org/10.1101/gr.849004

2. Liu Y, Sheng J, Fokine A, Meng G, Shin WH, Long F, et al. Structure and inhibition of EV-D68, a virus that causes respiratory illness in children. Science. 2015;347:71–4. PubMed https://doi.org/10.1126/science.1261962
